# Clinical Outcomes of Cataract Surgery in Patients with Sjögren’s Syndrome

**DOI:** 10.3390/diagnostics13010057

**Published:** 2022-12-25

**Authors:** Donghyeon Lee, Charm Kim, Kyeongjoo Lee, Jin Kwon Chung

**Affiliations:** Department of Ophthalmology, Soonchunhyang University Seoul Hospital, Seoul 04401, Republic of Korea

**Keywords:** cataract surgery, Sjögren’s syndrome, dry eye disease, superficial keratitis, ocular surface optimization, biometry, keratometry, visual acuity

## Abstract

This study compared the biometric accuracy and refractive outcomes, and ocular surface changes after cataract surgery in patients with Sjögren’s syndrome (SS, S group), non-SS dry eye patients (D group), and healthy controls (C group). The medical records of patients who underwent cataract surgery and met certain inclusion criteria were reviewed. In total, 167 eyes of 87 patients were enrolled. Refractive parameters were analyzed via optical biometry and combined ultrasound biometry and automated refractokeratometry. The mean absolute errors (MAEs), the uncorrected distance visual acuities (UDVAs), changes in the ocular staining score (OSS), and anterior chamber cell grades were compared for 12 months postoperatively. The S group evidenced more severe and persistent OSS exacerbation after cataract surgery; the OSS returned to baseline by 3 months postoperatively. The mean keratometric values showed a significant linear correlation. There was no significant intergroup difference in either the MAEs (*p* > 0.530) or anterior chamber inflammation (*p >* 0.436). The postoperative UDVA of the S group was poorer than that of the C group from 3 months postoperatively (*p <* 0.047) but not different from that of the D group (*p >* 0.311). With preoperative ocular surface optimization and optimal postoperative treatment of superficial keratitis, the refractive outcomes of SS patients were comparable to those of other groups and the postoperative UDVA was not inferior to that of non-SS dry eye patients.

## 1. Introduction

Sjögren’s syndrome (SS) is a chronic, multi-systemic autoimmune disease characterized by exocrine gland hypofunction caused by infiltration of inflammatory cells, triggering xerostomia and keratoconjunctivitis sicca (KCS) in about 95% of patients [1,2]. Patients with SS exhibit more severe dry eye symptoms, inflammatory reactions of the ocular surface, and high corneal and conjunctival staining scores compared to those without SS [3,4]. 

Tear film instability and the corneal erosion associated with SS dry eye significantly change the light pathway, and decrease the quality of vision and visual acuity, by increasing ocular aberrations, especially in high-order aberrations [5,6] Tear film instability and the corneal erosion also create errors when measuring the corneal power by keratometry for intraocular lens (IOL) power calculation and can result in residual refractive errors after cataract surgery [7].

Previous studies have reported exacerbation of the subjective symptoms and ocular surface disorders of patients after phacoemulsification prior to correction of various conditions [8,9,10]. Thus, ocular surface disease is associated with adverse outcomes after cataract surgery. Optimization of the ocular surface prior to cataract surgery may be important. As eyes with rheumatological disorders, including SS eyes, are more vulnerable to inflammation, vascular abnormalities, and dysregulation of immune homeostasis, cataract surgery may induce severe or long-lasting postoperative inflammation [11]. In this study, we evaluated whether optimal ocular surface preparation would improve preoperative biometric accuracy and compared refractive outcomes, uncorrected distance visual acuity (UDVA), and intraocular inflammation after cataract surgery in SS dry eyes compared to non-SS dry eyes and the eyes of normal healthy controls.

## 2. Methods

### 2.1. Study Design and Grouping

The medical records of patients who underwent phacoemulsification and IOL implantation by the same surgeon at the Department of Ophthalmology, Soonchunhyang University Seoul Hospital between 1 March 2015 and 29 February 2020 were retrospectively reviewed after approval was obtained from the Soonchunhyang University Hospital institutional review board (No. 2018-06-009). The study adhered to the relevant tenets of the Declaration of Helsinki and all patients provided written informed consent before cataract surgery. All patients were divided into three groups. The first was an S group of patients diagnosed with SS using the American-European Consensus Group criteria [12] or the 2016 American College of Rheumatology/European League Against Rheumatism (ACR/EULAR) criteria [1,13]. Such patients were regularly followed up and treated before cataract surgery to alleviate KCS. Ocular surface damage was assessed via fluorescein staining and patients with Oxford Scheme scores ≤2 on at least two consecutive visits were scheduled for preoperative ocular biometry and cataract surgery. The D group was composed of patients diagnosed with mild to moderate non-SS dry eye, using the DEWS I or II criteria, who had used artificial tears for at least 6 months [14,15]. The last group, the C group, included patients without dry eye symptoms scheduled for cataract surgery having normal tear break-up times (TBUTs > 10 s) and the Schirmer test results (>15 mm/5 min). Patients who were followed up for at least 12 months were included. To evaluate visual performance and the effect of superficial punctate keratitis (SPK) alone on refractive error after cataract surgery, all patients with ≤0.50 diopters (D) of corneal astigmatism and who received the same IOLs targeting the smallest myopic postoperative refraction were included. The exclusion criteria were the regular use of any topical eyedrops, except for dry eye treatment in the S and D groups; any prior ocular surgery; any macular or cicatricial ocular surface disease (e.g., ocular pemphigoid, conjunctival scarring, or chemical injury); any eyelid disease (e.g., moderate to severe blepharitis, any anatomical or neurological abnormality); the use of contact lenses; and/or an allergy to any medication prescribed.

### 2.2. Preoperative and Postoperative Examinations

All patients underwent complete ophthalmic examinations preoperatively, including measurement of UDVA which was tested from 3 m chart, the manifest refraction test, the Schirmer test without anesthesia, the TBUT, corneal fluorescein staining, tonometry, endothelial cell density measurement using a noncontact specular microscope (EM-3000, Tomey Corp., Nagoya, Japan), and a macular scan employing optical coherence tomography (Spectralis HRA-OCT, Heidelberg Engineering Inc., Heidelberg, Germany). Ocular surface damage was assessed using a slit lamp fitted with a cobalt blue filter and a yellow optical filter 1 min after instillation of a drop of 2% sterile fluorescein to the conjunctival sac. We used the Oxford Scheme to assess the ocular staining score (OSS); this ranged from 0 (absent) to 5 (severe) [16]. Biometric data were obtained using both optical biometry (IOLMaster 500, Carl Zeiss Meditec, Jena, Germany) and a combination of immersion-type ultrasound biometry (HiScan, Optikon Corp., Roma, Italy) and automated keratometry (KR 8900, Topcon Crop., Tokyo, Japan). The IOL powers were calculated using the Barrett Universal II formula that employed the biometric data yielded by the two methods. All ocular biometries were performed by a single experienced specialist who exercised particular care for patients with ocular surface conditions.

Routine postoperative examinations were scheduled at 1 week and 1, 3, 6, and 12 months after surgery. Ocular surface damage after surgery was assessed by a corneal specialist. Anterior chamber cells were evaluated using slit-lamp biomicroscopy and any postoperative inflammation was graded using the uveitis nomenclature working group system [17]. To compare the accuracy of IOL power prediction by the two biometric methods, we calculated mean absolute errors (MAEs).

### 2.3. Surgical Procedure and Postoperative Care

Standard phacoemulsification was performed after induction of topical anesthesia with 2% lidocaine. A 2.8 mm clear corneal incision was created on the axis of the steep corneal astigmatism using keratome knife (ClearCut, Alcon Surgical Laboratories, Fort Worth, TX, USA). A foldable IOL (AcrySof IQ SN60WF, Alcon Surgical Laboratories) was implanted in a capsular bag. The IOL power targeted the smallest myopic postoperative refraction. Patients were instructed to apply 0.3% gatifloxacin (Gatiflo, Handok, Seoul, Korea) and 0.5% loteprednol etabonate (Lotepro, Hanlim Co. Ltd., Seoul, Korea) eyedrops four times daily after surgery for 2 weeks. Patients with dry eye continued to use artificial tear substitutes, including 0.15% sodium hyaluronate (New Hyaluni, Taejoon Pharm. Co. Ltd., Seoul, Korea), 0.18% sodium hyaluronate (Kynex 2, Alcon Korea Ltd., Seoul, Korea), or 3% diquafosol (Diquas; Santen Pharm. Co. Ltd., Osaka, Japan) immediately after surgery. Additionally, patients with SS continued to use topical 0.05% cyclosporine A (Restasis, Allergan Inc., Irvine, CA, USA) from postoperative day 1.

### 2.4. Statistical Analysis

UDVA values were converted to logMAR values. Patient demographics were compared via one-way ANOVA and the χ^2^ test. We used a generalized estimating equation (GEE) after adjusting for inter-eye correlations, age, and sex to evaluate the target refractions, MAEs, anterior chamber cell grades, OSSs, and UDVAs of the three groups. The Bonferroni adjustment was employed for multiple comparisons. The differences in the MAEs derived using the two methods and the OSS changes after surgery were compared using the Wilcoxon signed rank test. The paired t-test was employed to compare the differences in mean keratometric values. Simple correlation analysis was used to derive correlations between the mean keratometric values afforded by the two devices. Required sample size was calculated with the values of MAE and UDVA based on the test power of 80% at the 0.05 significance level using ‘pwr’ package in R (version 3.6.1, R Core Team, Vienna, Austria). SPSS software (version 23, SPSS Inc., Chicago, IL, USA) was used for all statistical analyses, and *p* < 0.05 was taken to indicate statistical significance.

## 3. Results

The study included 87 patients (167 eyes) after applying the inclusion/exclusion criteria; all surgeries were uncomplicated. There were 26 patients (50 eyes), 30 (58), and 31 (59) in the S, D, and C groups, respectively. There were no significant intergroup demographic differences except in terms of sex (Table 1).

### 3.1. Ocular Surface Evaluation

**Figure 1:**
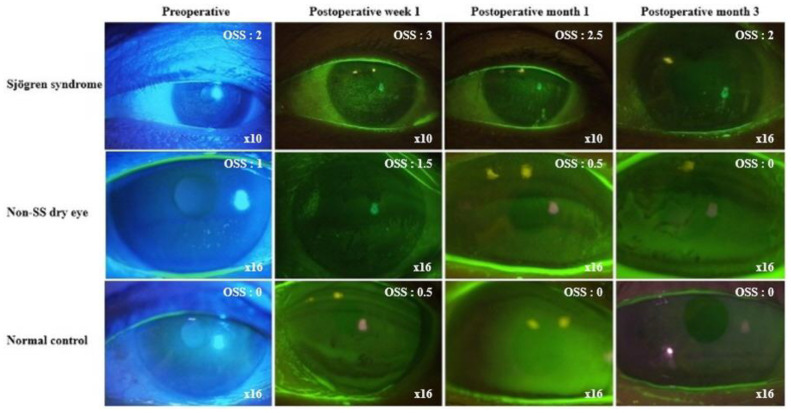
Representative slit-lamp findings in patients with Sjögren syndrome (SS), non-SS dry eye, and normal control before and after cataract surgery. Magnification of slit lamp photographs and ocular staining score (OSS) of each image are indicated. The SS patient showed a higher level of ocular staining score than the other two patients at every visit. Temporary exacerbation of corneal erosion was observed in all groups after cataract surgery. The SS patients had a longer recovery time.

The S group showed a significantly higher mean OSS than the D and C groups throughout the study period (*p* < 0.001). The D group had a significantly higher mean OSS than the C group throughout the study period, except at postoperative month 12. The OSSs revealed temporary exacerbations at postoperative week 1 in all groups. The values returned to baseline levels by postoperative month 1 in the D and C groups. However, that of the S group did not attain the baseline level until postoperative month 3 (Table 2, Figure 1). 

### 3.2. Refractive Outcomes

The preoperative mean IOLMaster keratometric values were higher than the automated refractokeratometry (ARK) values in all groups (*p* < 0.001). The mean values of the two methods evidenced a significant linear correlation. The Pearson correlation coefficients were >0.990 (*p* < 0.001) for all groups (Table 3). The preoperative target diopters did not significantly differ among the three groups regardless of whether IOLMaster (*p* = 0.615) or a combination of ultrasound biometry and ARK (*p* = 0.474) was used to derive the values. In the S group, the predicted target diopters of the two biometric methods did not significantly differ, but they did in the other groups (Table 4). The postoperative MAEs did not significantly differ among the groups at any time, regardless of the biometric measurement method. In the S group, the MAEs did not differ throughout the study when either method was used. The MAEs calculated via ultrasound biometry and ARK were lower at postoperative month 1 in the D group and at postoperative months 6 and 12 in the C group (Table 4). The UDVA improved after postoperative week 1 in all patients (*p* < 0.001) and remained stable up to 12 months postoperatively. The groups did not differ in terms of the mean UDVAs at 1 week and 1 month postoperatively. However, from postoperative month 3, the mean UDVA of the S group was poorer than that of the C group. The mean UDVAs of the S and D groups did not differ throughout the study period (Table 5).

### 3.3. Postoperative Inflammation

Anterior chamber inflammation was maximal in postoperative week 1 and then began to decline in all groups. No anterior chamber cells remained after postoperative month 3. There were no significant differences in anterior chamber cell grades among the groups either at postoperative week 1 (*p* = 0.436) or postoperative month 1 (*p* = 0.784).

## 4. Discussion

After cataract surgery, some patients are dissatisfied. They complain of suboptimal refractive outcomes or worsening of dry eye symptoms; these may indicate damage to the ocular surface. Increasing recognition of these issues suggests that preoperative ocular surface optimization is imperative [18,19]. SS patients with KCS are susceptible to such damage. Therefore, minimization of this issue prior to cataract surgery is imperative to ensure satisfactory outcomes and safety. We highlight the favorable clinical outcomes of cataract surgery in patients with well-controlled SS.

Patients in the S group received individualized preoperative treatments such as warm compression, lid hygiene advice, artificial-tear substitutes, topical steroids, cyclosporine, autologous serum, and/or punctal occlusion. Although only patients with OSSs ≤ 2 were included, the mean preoperative OSS was higher in the S group than in the other groups, attributable to the chronic nature of dry eye disease, which is characterized by tear-film instability, inflammation, and thus susceptibility to ocular surface damage [15]. SS is one of the most severe types of dry eye disease. The results are consistent with studies that have reported that SS dry eyes have higher ocular surface staining scores than non-SS dry eyes [4,20,21].

As reported in previous studies, the OSSs were exacerbated after phacoemulsification in all groups but recovered to the baseline level by 1 to 3 months postoperatively [10,22,23]. However, the S group experienced longer and more severe ocular surface damage than did the other groups. When an external stimulus activates sensory nerves at the ocular surface, a lacrimal gland reflex is triggered; the glands produce and secrete tears [24]. However, a clear corneal incision causes temporary damage to the sensory nerve, preventing the normal reflex for some time [25]. Given the enduring inflammation of SS dry eyes, the nociceptive terminals change, slowing regeneration of corneal axons injured during surgery [26,27]. Despite continuous individualized treatment after surgery, the slower OSS recovery of the S group seemed to be associated with reduced exocrine gland function and the negative effects of chronic inflammation.

Since the advent of optical biometry, many studies have compared keratometric values with those yielded by older devices and the measurements have been in agreement. Mehravaran et al. reported that the IOLMaster and Topcon automated refractometers were in best agreement with the manual Javal keratometer, the gold standard, and could be safely used instead of the latter to evaluate normal subjects in clinical settings [28]. However, tear-film instability, a central feature of dry eye, reduces the quality of corneal reflection and thus compromises keratometric readings. During preoperative cataract surgery planning, the keratometric values can vary on different visits by patients with dry eye, and in turn, the IOL power calculations can significantly differ [7]. For patients with SPK refractory to treatment, we sought to minimize the effect of keratometric variability on IOL power calculations. Although SPK remained after treatment in many patients in the S group, as the MAEs, which directly reflect the residual refractive errors, did not differ among the groups, we suggest that the keratometric values were relatively accurate even in SS patients with lower grade SPK.

As is already well-documented, in this study, the mean keratometric values measured by the IOLMaster were slightly higher than those measured via ARK in all groups [29]. The Pearson coefficients of the mean corneal powers measured by the two devices were >0.990 for all groups, thus similar to the 0.985 of a study that excluded patients with corneal disease [30]. Thus, if the SPK of SS is managed to some extent prior to biometry, the keratometric values yielded by different devices are significantly correlated; any difference is clinically insignificant.

The MAEs calculated using the two biometric methods did not significantly differ in the S group. Although significant differences were apparent at 1 month in the D group and at 6 and 12 months in the C group, all values were below 0.1 D, thus clinically insignificant. We found no significant intergroup differences in the MAE values of the two methods. Thus, the predictability of the postoperative refraction of SS patients with OSSs ≤ 2 after optimization of the ocular surface was comparable to that of normal patients, no matter which biometric method was used. Hsieh et al. reported that the IOLMaster MAE was 0.38 ± 0.28 D and the values of conventional ARK and contact acoustic biometry were 0.54 ± 0.37 D 1 month postoperatively [31]. In this study, IOLMaster MAEs were 0.30–0.36 D and those of the conventional method were 0.30–0.31 D 1 month postoperatively. Although the MAEs tended to increase over 12 months, they remained within 0.5 D regardless of the biometric method used or the study group. Thorough preoperative management of patients with dry eye improves postoperative refractive outcomes [19,32]. By proactively addressing ocular surface status, the IOL power calculations and the refractive outcomes became comparable to those of healthy subjects.

The S group exhibited a postoperative UDVA similar to that of the D group throughout the study, but the UVDA of the C group diverged from postoperative month 3. As the factors that might affect postoperative visual acuity—such as IOL prediction errors, postoperative anterior chamber inflammation, or retinal or optic nerve disease—did not significantly differ among the groups, the SPK may explains the difference in the postoperative UDVAs. Corneal surface irregularities generate HOA and reduce contrast sensitivity given the lack of a perfectly refractive surface [33,34]. Corneal surface damage is also associated with increased corneal backward light scattering that can trigger blurred vision and eye fatigue [35]. One study employed a functional visual acuity measurement system, and found that dry eyes with SPK evidenced significant visual deterioration compared to dry eyes without SPK, and that the severity of epithelial damage correlated significantly with changes in visual acuity [36]. Villarreal-Gonzalez et al. confirmed a negative correlation between the OSS and visual acuity in SS patients [37].

Previous studies have found that blood aqueous barrier breakdown occurs during surgery, attributable to direct trauma to the anterior uvea. The anterior chamber inflammatory response peaks immediately after surgery, followed by a continuous decline over the next month. Decreases in anterior chamber cell counts were also observed; grade 0 was attained in all patients by postoperative month 3. A study that employed laser flare photometry reported that baseline anterior chamber flare, a marker of blood aqueous barrier disruption, was higher in SS dry eyes and non-SS dry eyes than in normal controls [38]. Although the methods used to evaluate anterior chamber inflammation and the extent of SS differed, this study confirms that well-controlled patients with SS experience postoperative inflammation similar to that of other groups after uneventful cataract surgery.

To the best of the authors’ knowledge, this is the first comparative study to report the clinical outcomes of cataract surgery including UDVA and refractive outcomes in patients with SS. An upper bound to the OSS was set and it was found that biometric measurements were reliable and cataract surgery was safe in SS patients. No refractive errors were evident in SS patients meeting the OSS criterion. The results of this study will help surgeons inform SS patients who are considering cataract surgery about the postoperative ocular surface changes and the expected visual acuity outcomes.

This study had certain limitations. Non-SS dry eye was not classified by subtype and SPK treatment was not uniform. An absence of quantitative measurement to assess a patient’s subjective experience of dry eye symptoms before and after cataract surgery that could identify patients with a neuropathic component in their dry eye. As only SS patients with OSSs ≤ 2 were included, further studies in subjects with different extents of corneal damage are necessary.

## 5. Conclusions

After preoperative ocular surface optimization, there were no significant intergroup differences in refractive outcomes after cataract surgery. Although the postoperative UDVA of SS patients was poorer than that of non-dry eye patients), it was comparable to that of non-SS dry eye patients. Ocular surface optimization before cataract surgery is effective; this is essential to improve the clinical outcomes of patients with SS.

## 6. Value Statement

What was known:

- Ocular surface damage associated with dry eye reduces quality of vision and triggers errors in keratometric measurements that render the calculation of intraocular lens power inaccurate.
- Ocular surface optimization is required to ensure good refractive outcomes and patient satisfaction after cataract surgery; no prior study has focused on dry eye associated with Sjögren’s syndrome (SS).

What this paper adds:

- Management of superficial punctate keratitis in patients with SS requiring cataract surgery minimizes biometric and postoperative refractive errors.
- The uncorrected distance visual acuity of patients with well-controlled SS is comparable to that of non-SS dry eye patients for 12 months after phacoemulsification.

## Figures and Tables

**Table 1:** Patients Demographics.

	Sjögren Group	Dry Eye Group	Control Group	*p*-Value
Number (Patients [eyes])	26 (50)	30 (58)	31 (59)	
Age (years, Mean ± SD)	68.38 ± 8.80	68.03 ± 7.01	67.54 ± 6.61	0.840 *^a^*
Sex (Female/Male)	26/0	23/7	25/6	<0.001 *^b^*
Side (Right/Left)	25/25	29/29	29/30	0.995 *^b^*

*^a^* One-way ANOVA; *^b^* χ^2^ test.

**Table 2:** Preoperative and Postoperative OSSs.

	Sjögren Group		Dry Eye Group		Control Group		*p*-Value *^a^*
	Mean ± SD	*p*-Value *^b^*	Mean ± SD	*p*-Value *^b^*	Mean ± SD	*p*-Value *^b^*	
Preoperative	1.38 ± 0.83		0.59 ± 0.53		0.14 ± 0.39		<0.001
Postop. Week 1	2.43 ± 1.46	<0.001	0.93 ± 0.65	0.001	0.46 ± 0.62	0.001	<0.001
Postop. Month 1	1.86 ± 1.37	0.002	0.38 ± 0.62	0.063	0.15 ± 0.36	0.617	<0.001
Postop. Month 3	1.34 ± 1.14	0.922	0.45 ± 0.57	0.117	0.16 ± 0.37	0.564	<0.001
Postop. Month 6	1.12 ± 1.06	0.111	0.36 ± 0.52	0.019	0.07 ± 0.26	0.366	<0.001
Postop. Month 12	1.22 ± 1.17	0.214	0.29 ± 0.46	<0.001	0.16 ± 0.42	>0.999	<0.001

OSS = ocular staining score; Postop. = postoperative. *^a^* A generalized estimating equation after adjustment for age, sex, and inter-eye correlations to analyze differences between the three groups at each timepoint; *^b^* The Wilcoxon signed rank test was employed to compare the postoperative OSSs and preoperative OSSs.

**Table 3:** Mean Keratometry Values Measured by the IOLMaster and ARK.

	Sjögren Group	Dry Eye Group	Control Group
Mean K (D, Mean ± SD)			
IOLMaster	44.69 ± 1.12	44.51 ± 1.48	44.35 ± 1.25
ARK	44.57 ± 1.18	44.32 ± 1.44	44.19 ± 1.22
*p*-value *^a^*	<0.001	<0.001	<0.001
*r* (*p*-value)	0.990 (<0.001)	0.995 (<0.001)	0.992 (<0.001)

ARK = Automated refractokeratometer; D = Diopter; K = Keratometry value; *r* = Pearson correlation coefficient. *^a^* The paired t-test was used to compare the difference in mean K values measured by the two devices.

**Table 4:** Preoperatively Predicted Target Refractions and MAEs Calculated Using the Measurements of the IOLMaster and a Combination of Ultrasound Biometry and ARK.

	Sjögren Group			Dry Eye Group			Control Group			*p*-Value *^a^*	
	Mean ± SD		*p*-Value *^b^*	Mean ± SD		*p*-Value *^b^*	Mean ± SD		*p*-Value *^b^*		
	M	US + ARK		M	US + ARK		M	US + ARK		M	US + ARK
Target refraction (D)	−0.31 ± 0.19	−0.31 ± 0.17	0.489	−0.37 ± 0.19	−0.29 ± 0.21	<0.001	−0.37 ± 0.25	−0.32 ± 0.25	0.022	0.615	0.474
MAE Month 1 (D)	0.30 ± 0.26	0.31 ± 0.27	0.709	0.36 ± 0.22	0.30 ± 0.22	0.029	0.34 ± 0.30	0.31 ± 0.29	0.201	0.856	0.984
MAE Month 3 (D)	0.35 ± 0.30	0.35 ± 0.33	0.654	0.33 ± 0.23	0.33 ± 0.23	0.901	0.36 ± 0.31	0.33 ± 0.29	0.109	0.530	0.765
MAE Month 6 (D)	0.38 ± 0.32	0.37 ± 0.36	0.856	0.39 ± 0.24	0.39 ± 0.24	0.912	0.39 ± 0.31	0.34 ± 0.28	0.032	0.682	0.746
MAE Month 12 (D)	0.42 ± 0.37	0.39 ± 0.36	0.416	0.40 ± 0.27	0.39 ± 0.28	0.595	0.41 ± 0.32	0.36 ± 0.31	0.019	0.988	0.728

ARK = automated refractokeratometer; D = Diopter; K = keratometry value; M = IOLMaster; MAE = mean absolute error; US = Ultrasound biometry. *^a^* A generalized estimating equation after adjustment for age, sex, and inter-eye correlations to analyze differences between the three groups at each timepoint; *^b^* Wilcoxon signed rank test was employed to compare the values calculated by the measurements from the two methods at each timepoint.

**Table 5:** Preoperative and Postoperative UDVAs (Log MAR).

		Mean ± SD		*p*-Value *^a^*		*p*-Value *^a^*	
	Sjögren Group	Dry Eye Group	Control Group		S vs. D	S vs. C	D vs. C
Preoperative	0.43 ± 0.33	0.34 ± 0.19	0.43 ± 0.31	0.109	0.330	>0.999	0.217
Postop. Week 1	0.11 ± 0.09	0.10 ± 0.09	0.09 ± 0.10	0.555	>0.999	>0.999	0.845
Postop. Month 1	0.11 ± 0.09	0.08 ± 0.08	0.07 ± 0.08	0.170	>0.999	0.182	>0.999
Postop. Month 3	0.09 ± 0.10	0.08 ± 0.06	0.05 ± 0.06	0.017	>0.999	0.034	0.179
Postop. Month 6	0.09 ± 0.09	0.07 ± 0.06	0.05 ± 0.05	0.046	0.422	0.047	0.757
Postop. Month 12	0.11 ± 0.09	0.07 ± 0.06	0.05 ± 0.06	0.007	0.311	0.009	0.342

C = control group; D = dry eye group; Postop. = postoperative; S = Sjögren group; UDVA = uncorrected distance visual acuity. *^a^* A generalized estimating equation after adjustment for age, sex, and inter-eye correlations to analyze differences between the three groups at each timepoint.

## Data Availability

The data presented in this study are available in the article.
